# Lost without a cause: time to embrace causal thinking using Directed Acyclic Graphs (DAGs)

**DOI:** 10.1186/s12966-023-01545-8

**Published:** 2023-12-11

**Authors:** Jelle Van Cauwenberg, Annick De Paepe, Louise Poppe

**Affiliations:** 1 Department of Public Health and Primary Care, Ghent University, Ghent, Belgium; 2 Research Foundation Flanders, Brussels, Belgium; 3 Department of Experimental Clinical and Health Psychology, Ghent University, Ghent, Belgium

The primary focus of behavioral nutrition and physical activity research is to inform policies and practices targeting changes in individuals’ physical activity and nutrition behaviors. To effectively change these behaviors, knowledge about the factors that are causally affecting these behaviors is crucial. Randomized controlled trials (RCTs) are considered the gold standard to infer causality, but they are often unfeasible or unethical to conduct to address our research questions. Therefore, only relying on RCTs to infer causality would exclude addressing a range of research questions that are relevant to our research field. As a result, we often need to turn to quasi-experimental and observational studies to gain insight into these causal effects.

Early on in our scientific training we learn that ‘correlation does not imply causation’ and that quasi-experimental and observational studies cannot prove causation. Therefore, in non-randomized studies causal language is purposefully avoided when formulating our study aims [4]. Nevertheless, in the discussion section, recommendations for policy and future interventions, which do rely on causal assumptions, are often formulated. In this commentary we argue that the avoidance of causal thinking may lead to biased results and inadvertently hinders progress in the field. We argue to embrace causal thinking by being explicit and transparent about the causal aims of our research. We introduce Directed Acyclic Graphs (DAGs) to inform study design and/or analysis and to discuss under which assumptions our results can be interpreted causally.

## The importance of thinking causally

Refraining from causal language and, therefore, from causal thinking, is potentially harmful. A striking example is provided by an observational study examining factors associated with COVID-19-related death among 17 million people [17]. Within this study, all potential risk factors (ranging from age to health behaviors and comorbidities) were included simultaneously in one regression model, without carefully thinking about the underlying causal structure. As long as the resulting model is merely used for prediction purposes, this is not a problem. However, as soon as individual regression coefficients are being interpreted, this may result in biased and sometimes very counterintuitive results. Williamson et al. [17], for example, found that the COVID-19 risk was lower for current smokers as opposed to ex-smokers or people who never smoked. Although the authors indicated that their findings should not be interpreted causally, readers and sometimes the authors themselves did interpret the individual variables as causal [15]. As a result, policies based on these incorrect causal interpretations were implemented, which potentially increased the risk for COVID-19 among vulnerable populations. Giving a causal interpretation to regression estimates for covariates included in the same regression model is known as the mutual adjustment or Table 2 fallacy [16] and is very prevalent in health promotion publications, including papers written by the authors of this commentary.

## Directed Acyclic Graphs (DAGs)

Causal inference within epidemiology has been hugely influenced by a set of seminal causal criteria proposed by Sir Austin Bradford Hill [5]. However, Hill himself stated that these criteria are ‘viewpoints’ rather than strict criteria and that none of them should be taken as hard-and-fast rules of evidence that must be obeyed to speak about cause and effect. A more recent approach on causal inference is provided by the potential outcomes framework [2, 9]. This framework posits that a true causal effect is the difference between the observed outcome when the individual was exposed and the unobserved potential outcome had the individual not been exposed, all other things being equal. Because the unobserved potential outcome of an individual cannot be known, researchers often compare the average outcomes of exposed and unexposed groups. Application of this framework requires researchers to consider (amongst other criteria) the exchangeability of both groups, or in other words, whether the unexposed group would have the same risk of the outcome as the exposed group had they also been exposed. In order for this to hold, all variables that influence both the exposure and the outcome (i.e. confounders) should be controlled for. DAGs were developed within this framework and provide a tool to identify confounders (and other potential sources of bias). How DAGs relate to Bradford Hill’s ‘viewpoints’ is described in a paper by Shimonovich and colleagues [11].

**Fig. 1:**
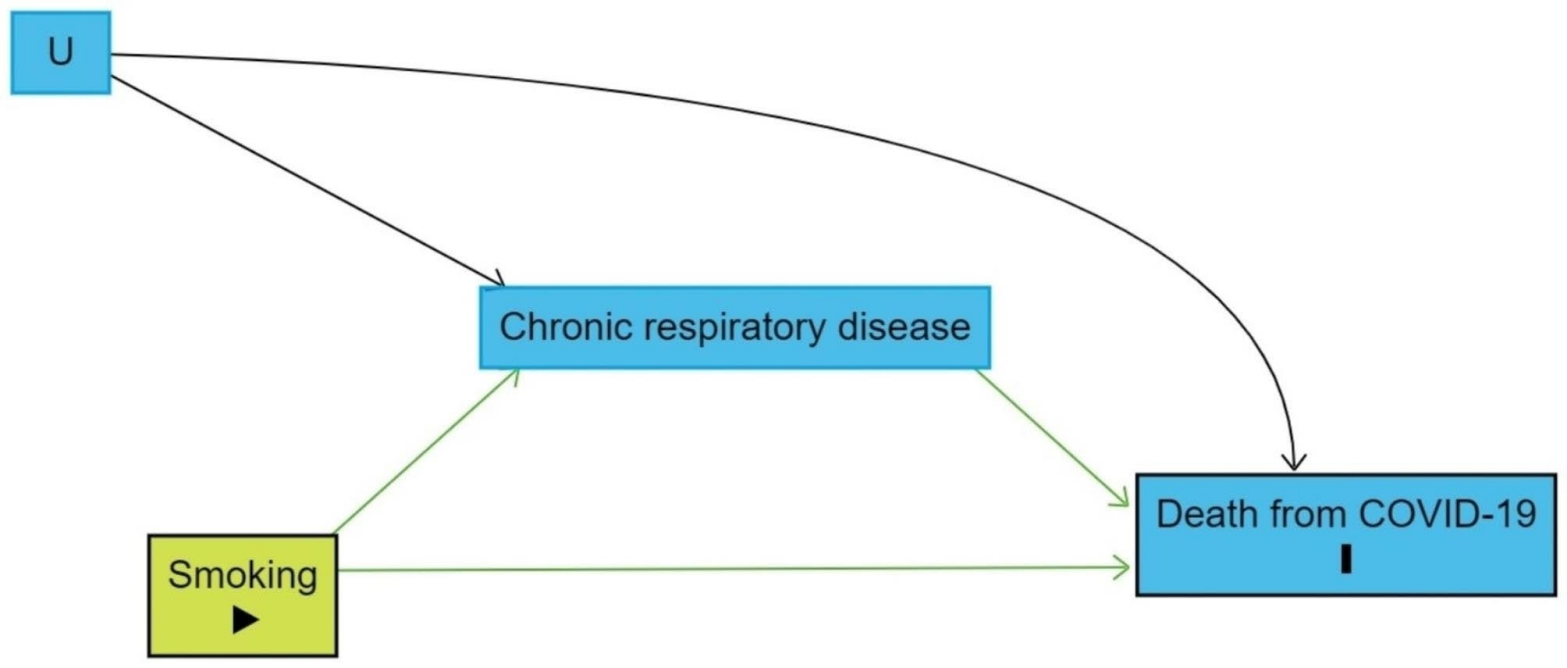
DAG created in ‘dagitty’ [13] for the effect of smoking on death from COVID-19, mediated by chronic respiratory disease, with confounding by unmeasured variables (U)

DAGs are schematic representations, developed based on expert knowledge, about the hypothesized causal relationships between the involved variables and can be used to identify confounders, mediators and colliders. This information can guide study design and statistical analysis to decide under which assumptions causal conclusions can be made based on (quasi-) experimental and observational data. In addition, their use promotes transparency by clearly and graphically presenting the a priori assumptions about the causal relationships involved. These assumptions can then be scrutinized in future studies facilitating cumulative research. For example, for the effect of smoking on death by COVID-19 introduced in the previous section, the causal diagram presented in Fig. 1 could illustrate what happened. The prediction model of Williamson et al. [17] did not only include smoking as a predictor, but also chronic respiratory disease, which is a mediator in the effect of smoking on death from COVID-19. As a result the estimate for smoking only takes into account the direct effect of smoking on death from COVID-19 and dismisses the indirect effect via chronic respiratory disease, leading to an underestimation of the total causal effect of smoking on death from COVID-19. Moreover, the effect estimate is also biased by a spurious pathway via unmeasured confounders (U), which could, for example, include a gene or air pollution that are influencing both risk for chronic respiratory disease and death from COVID-19. Without adjusting the model for chronic respiratory disease, these unmeasured confounders would not bias the estimate for smoking. However, when applying the ‘rules’ underlying DAGs, it becomes clear that adjusting for chronic respiratory disease results in opening a spurious pathway along these unmeasured confounders that biases the estimate for smoking.

When DAGs are developed before data collection, they can also provide important information on which measurements should definitely be performed and which not. This may shorten our endless lists of measurements and reduce participant burden. Within causal inference, methods have also been developed to handle missing data, selection bias, mediation analysis, and composite and compositional data [1, 6, 14], which are topics that are highly relevant for contemporary health promotion research. Nevertheless, this causal inference framework has not been widely embraced yet within health promotion research. For example, since its inception, only 22 papers published in IJBNPA have used a directed acyclic graph (DAG) to guide their study design and/or statistical analysis.

## Barriers for adopting causal thinking by using DAGs

The authors of this commentary have provided several introductory workshops of causal reasoning and the use of DAGs targeting health promotion and public health researchers including two workshops at the Annual Meeting of the International Society of Behavioral Nutrition and Physical Activity. During these workshops several barriers for adopting causal thinking by using DAGs were raised. First, it was raised that creating the perfect DAG is beyond the scope of researchers’ capacities. Hence, one might argue that residual confounding and biased results are inevitable without randomization. The authors agree that it is not possible to create a perfect DAG. However, when presenting a carefully constructed DAG, one is at least transparent about the underlying assumptions and future research is informed about which variables should definitely be measured and adjusted for in the design and analysis. Additionally, several methods exist to conduct sensitivity analyses for unmeasured confounders [7], together with R packages to implement these methods (e.g. tipR package: https://cran.r-project.org/web/packages/tipr/tipr.pdf). Furthermore, negative controls can be used to detect suspected and unsuspected sources of confounding [10]. Second, researchers often point out that they suspect the relationship between two variables of interest to be bidirectional. Since DAGs are ‘acyclic’ they do not include feedback between variables since the cause always has to precede the effect. A first solution may be to develop multiple DAGs representing different directions of the causal relationships and compare the results from the statistical models informed by the different DAGs. A second solution is to incorporate multiple time points within a DAG to depict the influence of the cause measured at time point one on the effect measured at time point two, which in turn influences the cause measured at time point three etc. [8]. Finally, it was raised that causal reasoning and DAGs might be another ‘research hype’. However, both the theoretical and the applied research fields of causal inference have evolved steadily and the use of DAGs has gradually increased over the past years in various fields of research [12].

## How to start embracing causality?

As already pointed out above, one of the most useful causal inference tools that can guide study design and analyses are DAGs. An excellent introduction to DAGs is given by Miguel Hernán in a free EdX course entitled ‘Causal diagrams: Draw your assumptions before your conclusions’ (https://www.edx.org). The book ‘Causal inference: what if’ starts on an introductory level and increases complexity throughout the book and is freely available on: https://www.hsph.harvard.edu/miguel-hernan/causal-inference-book/. A systematic review on the use of DAGs in applied health research including several recommendations for their use is provided by Tennant et al. [12]. Finally, a very useful tool to start drawing your own DAGs is ‘dagitty’ that can be used in a browser-based environment (https://dagitty.net/) as well as with an R package [13]. Once you have created your DAG it is highly recommended to include the DAG in your paper, such that you are transparent about your assumptions. We are convinced that DAGs can increase the transparency and robustness of scientific research within the field of behavioral nutrition and physical activity. Nevertheless, it is worth mentioning that we have only introduced one framework (the potential outcomes framework), and one tool based on this framework (i.e., DAGs), but that there are several ways to embrace causality in research. As all methods have their own limitations, causal triangulation of results across methods, with different sources of potential bias, is warranted [3].

## Conclusion

RCTs remain the ‘gold standard’ to infer causal effects, but for many of our research questions conducting an RCT is unfeasible or unethical. A choice then emerges: shying away from these questions or in contrast embracing causal thinking within observational and quasi-experimental studies. We argue that the importance of addressing these questions may not be undermined and hence we should embrace causal thinking. We have proposed DAGs as a tool to visualize the causal structure of your data and to determine under which assumptions causal effects can be identified. We strongly belief that this is the avenue to follow to advance the field of behavioral nutrition and physical activity. Let’s be transparent about our research aims and embrace causal thinking!

## Data Availability

Not applicable.

## List of abbreviations

DAG: Directed Acyclic Graph
RCT: Randomized Controlled Trial
